# Nutrient Transporter Gene Expression in the Early Conceptus—Implications From Two Mouse Models of Diabetic Pregnancy

**DOI:** 10.3389/fcell.2022.777844

**Published:** 2022-04-11

**Authors:** Claudia Kappen, Claudia Kruger, Sydney Jones, J. Michael Salbaum

**Affiliations:** ^1^ Department of Developmental Biology, Pennington Biomedical Research Center, Louisiana State University System, Baton Rouge, LA, United States; ^2^ Regulation of Gene Expression, Pennington Biomedical Research Center, Louisiana State University System, Baton Rouge, LA, United States

**Keywords:** neural tube defect, visceral endoderm, yolk sac, implantation site, mid-gestation mouse embryos

## Abstract

Maternal diabetes in early pregnancy increases the risk for birth defects in the offspring, particularly heart, and neural tube defects. While elevated glucose levels are characteristic for diabetic pregnancies, these are also accompanied by hyperlipidemia, indicating altered nutrient availability. We therefore investigated whether changes in the expression of nutrient transporters at the conception site or in the early post-implantation embryo could account for increased birth defect incidence at later developmental stages. Focusing on glucose and fatty acid transporters, we measured their expression by RT-PCR in the spontaneously diabetic non-obese mouse strain NOD, and in pregnant FVB/N mouse strain dams with Streptozotocin-induced diabetes. Sites of expression in the deciduum, extra-embryonic, and embryonic tissues were determined by RNAscope *in situ* hybridization. While maternal diabetes had no apparent effects on levels or cellular profiles of expression, we detected striking cell-type specificity of particular nutrient transporters. For examples, Slc2a2/Glut2 expression was restricted to the endodermal cells of the visceral yolk sac, while Slc2a1/Glut1 expression was limited to the mesodermal compartment; Slc27a4/Fatp4 and Slc27a3/Fatp3 also exhibited reciprocally exclusive expression in the endodermal and mesodermal compartments of the yolk sac, respectively. These findings not only highlight the significance of nutrient transporters in the intrauterine environment, but also raise important implications for the etiology of birth defects in diabetic pregnancies, and for strategies aimed at reducing birth defects risk by nutrient supplementation.

## Introduction

Diabetes of the mother in early pregnancy is a well-known risk factor for heart defects and neural tube closure defects in the offspring (Molsted Pedersen et al., 1964; Kucera, 1971; Mills, 1982). In rodent models of diabetic pregnancy, the incidence of structural birth defects was shown to be reduced after supplementation of various micronutrients, among them Vitamin C, Vitamin E, Lipoic acid, Arachidonic Acid, and Folic Acid (Goldman et al., 1985; Piddington et al., 1996; Sivan et al., 1996; Viana et al., 1996; Reece and Wu, 1997; Siman and Eriksson, 1997; Wiznitzer et al., 1999; Cederberg et al., 2001; Wentzel and Eriksson, 2005; Wentzel et al., 2005; Reece et al., 2006; Oyama et al., 2009; Sugimura et al., 2009). We previously demonstrated that Folic Acid supplementation also leads to decreased frequency of neural tube defects in a genetic model of diabetes (Salbaum et al., 2015), the non-obese diabetic mouse strain (Leiter, 1989). Furthermore, we demonstrated that composition of the maternal diet can modulate the risk for neural tube defects in Streptozotocin-induced diabetic mouse pregnancies (Kappen et al., 2011). These evidences highlight the importance of nutrient availability for susceptibility to abnormal development under conditions of maternal diabetes.

The generally accepted pathogenic culprit in diabetic pregnancies is hyperglycemia (Cockroft and Coppola, 1977; Freinkel, 1988; Eriksson et al., 1991), consistent with findings of lower birth defect incidence in infants born to mothers with good glycemic control. However, even with optimal control and micronutrient supplementation, a substantial risk for birth defects remains (Evers et al., 2004; Banhidy et al., 2011; Agha et al., 2016; Nasri et al., 2018), paralleling the observations in rodent models that supplementation cannot completely suppress the outcomes of adverse exposure. In addition to hyperglycemia, human type I diabetes is also characterized by hyperlipidemia in the mother in early pregnancy (Reece et al., 1994a; Basu et al., 2012; Barrett et al., 2014), and in diabetic rats, increased triglyceride and fatty acid concentrations were found in the circulation (Shafrir and Khassis, 1982; Eriksson et al., 1991; Martin and Herrera, 1991). Thus, macronutrient availability is also altered in diabetic pregnancies, prompting us to investigate whether potential changes in the expression of nutrient transporters could account for the adverse outcomes of diabetic pregnancies.

Specifically, we focused on expression of genes in the Slc2a glucose transporter family, given association of several genes within this family to embryonic (Slc2a1/Glut1, Wang et al., 2006; Slc2a3/Glut3, Ganguly et al., 2007; Slc2a9/Glut9, Preitner et al., 2009) or pre-weaning (Slc2a2/Glut, Gulliam et al., 1997) lethality and intrauterine growth restriction (Slc2a3/Glut3; and Slc2a4/Glut4, Katz et al., 1995). Transporters encoded by this gene family differ in their specificity and selectivity for hexose molecules, and can also transport glucosamine, ascorbate, myo-inositol and urate, and in their tissue-specific expression patterns and localization within cells (Mueckler and Thorens, 2013). A second focus of the present study was on genes encoding fatty acid transporters. Five cell-surface-associated members of the Slc27a family have been identified, and deficiency of Slc27a4/Fatp4 results in pre- and post-natal lethality (Herrmann et al., 2003), whereas absence of Slc27a1/Fatp1 (Kim et al., 2004), and Slc27a5/Fatp5 (Doege et al., 2006) in mice alters their metabolism, as does deficiency of the fatty acid transporter CD36 (Febbraio et al., 1999). The lack of carnitine-palmitoyl transferases from the outer mitochondrial membrane results in embryonic lethality (Cpt1a, Nyman et al., 2005; Cpt1b, Ji et al., 2008) or reduced body weight (Cpt1c, Wolfgang et al., 2006), whereas deficiency of Cpt2 (from the inner mitochondrial membrane is associated with pre-weaning lethality and multiple anatomical alterations. As with glucose transporters, substrate specificity and selectivity varies, as do tissue- and cell-type-specific expression patterns. Our results from early pregnancy time points that precede the pathogenesis of neural tube closure defects are interpreted relative to their ramifications for the pathogenic mechanisms underlying defective neural tube closure in diabetic pregnancies.

## Materials and Methods

### Animals

Mice of the FVB/N strain were purchased from Charles River Laboratories, NOD mice were purchased from The Jackson Laboratory. Blood glucose levels in NOD females over the age of 12 weeks were monitored weekly; once the levels were greater than 250 mg/dl, the females were considered diabetic, and were mated to normoglycemic NOD male mice. Hyperglycemia was induced in FVB/N females after 8 weeks of age by Streptozotocin injection as described previously (Kappen et al., 2011; Salbaum et al., 2011; Kappen et al., 2012; Kappen et al., 2019). All mice were housed in specific pathogen-free conditions, and were fed Purina 5,001 chow diet. All animal husbandry was carried out in strict accordance with the recommendations in the Guide for the Care and Use of Laboratory Animals of the National Institutes of Health of the United States of America, covered by protocols approved by the Institutional Animal Care and Use Committee of the Pennington Biomedical Research Center, an entity in the Louisiana State University System in Baton Rouge, LA.

### RNA Isolation

RNA from individual mouse decidua (4–7 independent pregnancies from NOD and 3 different pregnancies from FVB the strain) at E7.5 and E8.5 was isolated using TRIzol Reagent (Life Technologies) according to the manufacturer’s instructions. In short, tubes containing the decidua in 1 ml TRIzol were thawed at room temperature, 200 μL chloroform was added, and the contents were mixed by vortexing for 10 s, before incubating at room temperature for another 5 min. After centrifugation at 15,300 x *g* for 15 min at 4°C, the aqueous layer was carefully transferred to a new set of tubes and 5 μg glycogen (Thermo Fisher Scientific), 500 μL Isopropanol (Fisher Scientific), and 10 μL 8M LiCl (Sigma-Aldrich) were added to assist with precipitation at −20°C overnight. The next morning, centrifugation occurred at 21,000 x *g* for 20 min at 4°C; the pellet was washed using 500 μL 70% ethanol. After centrifugation at 15,300 x *g* for 10 min at 4°C all ethanol was removed and the pellet allowed to air dry. The RNA pellet was dissolved in 50 μL nuclease-free water (Ambion) and incubated at 55°C for 15 min. RNA integrity was measured using the RNA 6000 Nano chip on the Agilent 2,100 Bioanalyzer (Agilent Technologies) which yielded RNA integrity numbers (RIN) of 6.8 and above for all investigated samples.

### Reverse Transcription

The High-Capacity cDNA Reverse Transcription Kit with RNase inhibitor (Applied Biosystems) was used to reverse transcribe 1 μg RNA from each sample into cDNA. In brief, the 20 μL reaction consisted of 10 μL 2X RT Master Mix and 10 μL RNA sample. The Master Mix contained 2.0 μL 10X RT Buffer, 0.8 μL 25X dNTP Mix (100 mM), 2.0 μL 10X RT Random Primers, 1 μL MultiScribe Reverse Transcriptase, 1 μL RNase Inhibitor and 3.2 μL Nuclease-free water. The thermal cycling conditions were as follows: 10 min at 25°C, 120 min at 37°C, 5 min at 85 and 4°C. Synthesized cDNA was transferred into a new tube and 80 μL nuclease-free water was added to a final concentration of 10 ng/μL for each sample.

### Primers

Gene sequences were downloaded from the Ensembl genome browser and Primer Express Software v3.0 (Applied Biosystems) was used in its default settings for primer pair design. Where possible, the amplicon was designed to span across an exon-exon boundary to exclude amplification from potential contamination with genomic DNA. The locations and sequences of primers have been published (Kappen et al., 2019). Synthesis of the primer sets was carried out by IDT (Integrated DNA Technologies, Coralville, IA). Primers were reconstituted with low-TE (Thermo Fisher) to a concentration of 100 μM and diluted with nuclease-free water to a working solution with a 10 μM concentration.

### Quantitative Real-Time PCR

Quantitative real-time PCR was performed on the 7900HT Sequence Detection System (Applied Biosystem) with default settings, including dissociation curves for each assay. Using the epMotion 5,075 (Liquid Handling Robot, Eppendorf), the PCR reactions were set up in a 384-well plate (Applied Biosystems) with 1.6 ng cDNA per reaction in a total volume of 10 μL per reaction, consisting of 3 μL cDNA, 5 μL iTaq Universal SYBR Green Supermix containing ROX as passive reference dye (Bio Rad), 1.8 μL water, 0.1 μL forward primer and 0.1 μL reverse primer. The thermal cycling reaction started with 2 min at 50°C and 10 min at 95°C for optimal DNA polymerase activation. PCR reactions consisted of a denaturation step of 15 s at 95°C, annealing and extension for 1 min at 60°C, for a total of 40 cycles. Reactions were run in triplicates, including no-template controls (water instead of cDNA) for each gene. Analyses were performed using six individual decidua from the FVB strain and twelve decidua from the NOD strain per condition and time point.

The comparative CT method (2^−ΔΔCT^) was used for relative quantification of gene expression (User Bulletin #2, Applied Biosystems). For each primer set, the actual amplification efficiency (AE) across all samples was calculated and implemented in the formula to calculate Fold-changes as described before (Kappen et al., 2012). Polymerase epsilon 4 (Polε4) was used as reference gene for normalization to derive ΔCt values. For each primer pair, the amplification efficiency was calculated by applying the formula: (Rncycle (n)/(Rncycle (n-1) over three consecutive cycles, starting at the determined Ct value in the geometric phase.

### Statistical Analysis

Statistical analysis of comparisons between conditions and strains was conducted using t-tests, assuming unequal variances and requiring two-sided *p*-values. *p*-values smaller than 0.05 were considered significant. DataAssist software (Applied Biosystems) was used to identify possible outliers among triplicate measurements.

### Histology

Individual decidua at E8.5 from normal and diabetic pregnancies were stored in 10% formalin until processed in the Tissue-Tek VIP instrument (Sakura Finetek USA, Inc., Torrance, CA, United States), and afterwards were paraffin-embedded with the Embedder Histocentre 3 (Thermo). Serial sections of 5 μm paraffin-embedded tissues were prepared using the Leica RM2255 fully automated rotary microtome (Leica Biosystems Inc.). Sections were applied onto Superfrost Plus Microscope Slides (Fisher Scientific) and stored in slide boxes until use. Cover-slipped slides were scanned in bright field settings using the NanoZoomer Digital Pathology (NDP) slide scanner (Hamamatsu, Bridgewater, NJ, United States).

### *In situ* Hybridization

To localize genes of interest in decidua derived from normal and diabetic pregnancies we used the RNAscope 2.5 HD Detection Kit (RED) (Advanced Cell Diagnostics, Hayward, CA, United States). Their double Z target RNA-specific oligonucleotide probes are designed to identify single RNA transcripts per cell. We followed the manufacturer’s protocol made available under www.acdbio.com/support. In short, after sections were deparaffinized and pretreated, hybridization to target RNA, and multi-step signal amplification were followed by a final detection step using Fast Red. The following adjustments were made to the vendor-supplied protocol: the pretreatment with Protease Plus was shortened to 15 min, counterstaining of slides was performed using Hoechst 3,342 (Invitrogen), and ProLong Gold antifade reagent with DAPI (Invitrogen) was used for mounting. Every experiment included probes for Cyclophilin B (Ppib) as a positive control and the bacterial gene dapB as a negative control.

### Validation of RNAscope Probes

Glycerol stocks for bacteria containing Slc2a1/Glut1, Slc2a2/Glut2, Slc2a3/Glut3, and Baz1b cDNA plasmids were obtained from Open Biosystems (Huntsville, AL, United States) and transferred onto LB plates using the appropriate antibiotics to obtain single colonies, which were then cultured. Plasmid DNA was extracted using the Zyppy plasmid miniprep kit (Zymo Research, CA, United States). Identity of each plasmid DNA was confirmed by Sanger sequencing. Extracted linearized (EcoRV) plasmid DNA was denatured at 98°C for 5 min and cooled down on ice-water. Droplets of 0.5 μL (2.5 and 0.5 ng DNA) of each plasmid were transferred onto Superfrost Plus Microscope Slides, and first dried at room temperature, before an incubation for 2hrs on a hot plate set to 80°C. Using RNAscope probes for Slc2a1/Glut1, Slc2a2/Glut2, and Slc2a3/Glut3, the above-mentioned protocol for *in situ* hybridization was followed as described (no pretreatment of the sample). Baz1b plasmid DNA served as negative control. Slides were cover-slipped with ProLong Gold antifade reagent containing DAPI and scanned using the NanoZoomer Digital Pathology (NDP) instrument.

### Next-Generation Sequencing Datasets

Transcriptomics data for embryos and yolk sac samples from next-generation RNAseq experiments had been generated previously (J.M. Salbaum et al., manuscript in preparation). Briefly, embryos and yolk sacs were dissected at the respective developmental stage from conceptuses of normoglycemic and diabetic pregnant dams of the non-obese diabetic (NOD) mouse strain (The Jackson Laboratory). Embryos and associated yolk sacs were isolated at embryonic day E8.5 and staged according to the presence of somite pairs, and individually subjected to the RNAseq Protocol. Results were expressed as normalized counts, with normalization relative to the size of each sequencing library, as implemented in DESeq2 (Love et al., 2014). The transcriptomics dataset analyzed in this study has been deposited in the Gene Omnibus database (accession number GSE197396).

## Results

**FIGURE 1:**
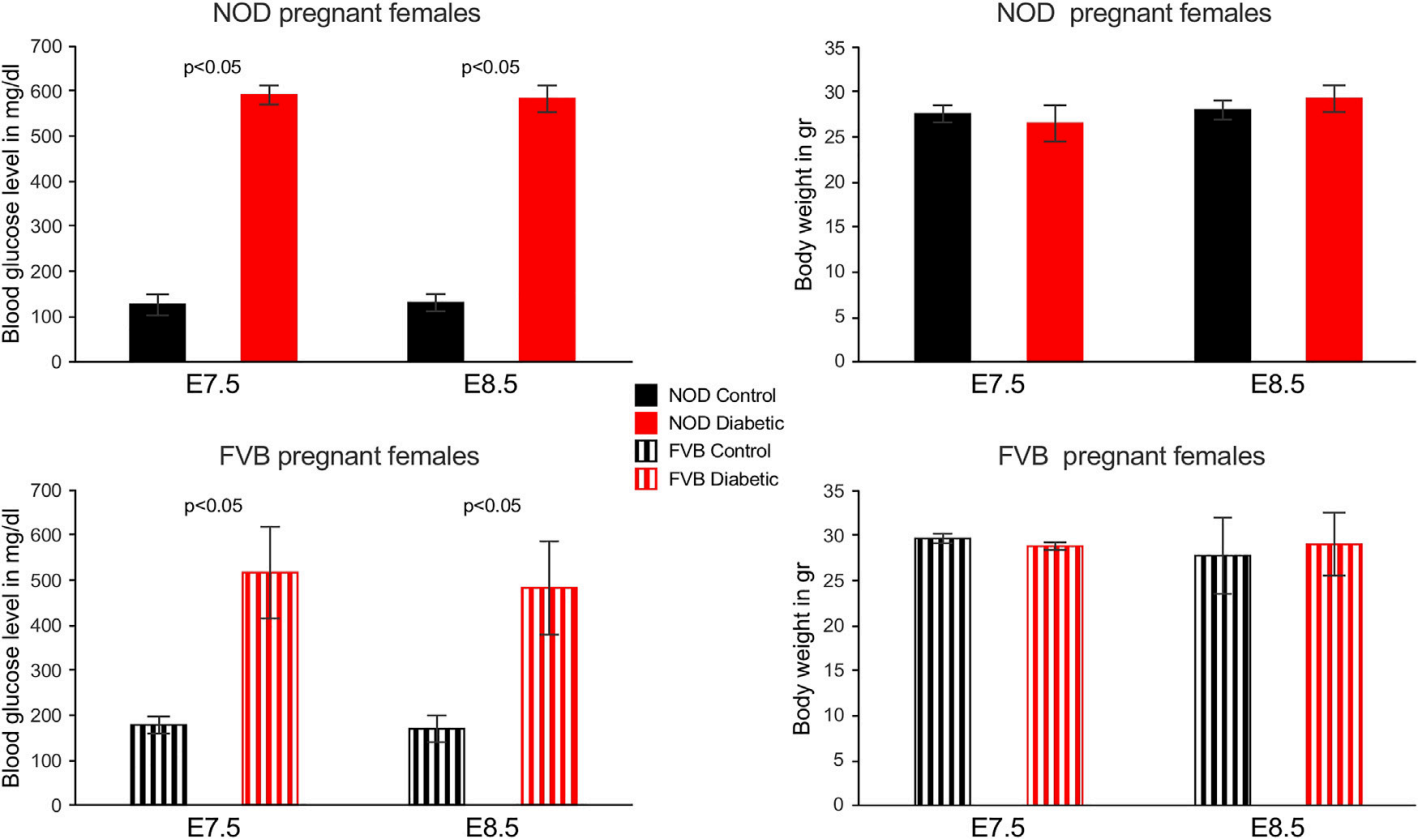
Blood glucose levels and weights of pregnant mouse dams. Blood glucose levels were measured weekly in NOD females and in FVB females after Streptozotocin administration, on the day of mating, and again at the time of sacrifice. All diabetic females exhibited glocuse levels higher than 360 mg/dl at the time of sacrifice, in cases where readings exceeded the scale of the glucometer, the value was set to 600 mg/dl (3 out of 5 NOD dams at E7.5, 4 out of 6 NOD dams at E8.5, one out of 3 FVB dams at E7.5). Weights of dams did not significantly differ between groups or time points.

Two mouse models of maternal diabetes in pregnancy were employed in this study: 1) the non-obese diabetic (NOD) strain in which females become hyperglycemic spontaneously between 12 and 18 weeks of age, and 2) FVB mice in which hyperglycemia was induced by administration of Streptozotocin; females with blood glucose levels above 250 mg/dl were considered diabetic/hyperglycemic and then mated to normoglycemic males of the respective strains to establish pregnancies. Depicted in Figure 1 are blood glucose levels and weights of pregnant dams at the time point of sacrifice. Blood glucose levels were consistently high in diabetic dams, but dam weights or litter sizes were not significantly different.

**FIGURE 2:**
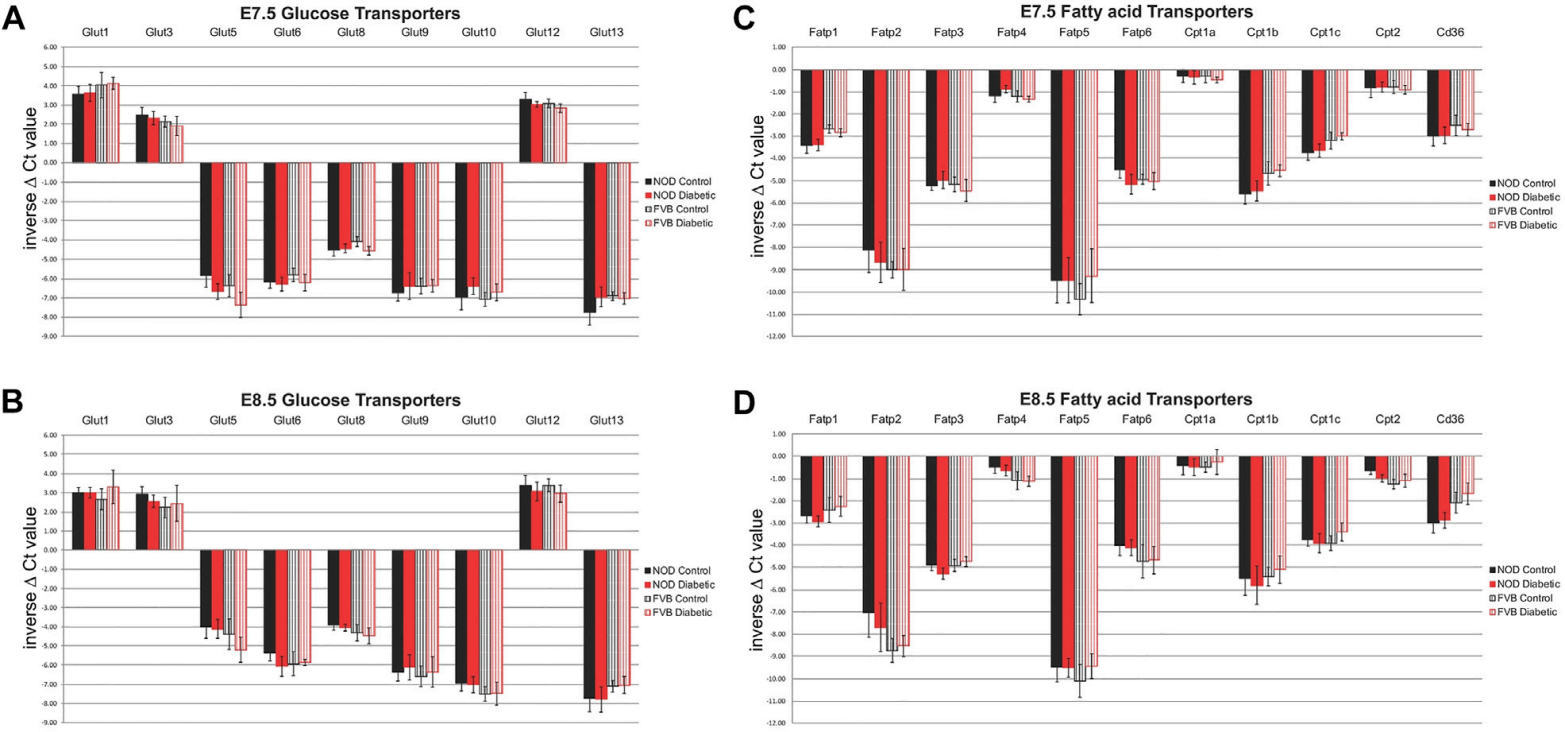
Expression of Glucose and Fatty Acid Transporters in decidua as measured by quantitative RT-PCR. Panels **(A)** and **(B)**: glucose transporters at E7.5 **(A)** and E8.5 **(B)** days of gestation; Panels **(C)** and **(D)**: fatty acid transporters at E7.5 **(C)** and E8.5 **(D)** days of gestation. Results are graphed as inverse ΔCt values so that the highest expression levels are depicted at the top of each figure. For FVB pregnancies (striped bars), each group consisted of 6 individual decidua (*n* = 6), from at least three independent pregnancies, for NOD pregnancies (solid bars), each group consisted of 12 individual decidua (*n* = 12), from at least 6 independent pregnancies, respectively. Samples from diabetic dams are depicted in red, samples from normoglycemic controls are shown in black. Expression levels were not significantly different between strains, nor when compared between metabolic conditions.

To measure nutrient transporter expression in maternal decidual tissue, individual implantation sites were isolated at 7.5 (E7.5) and 8.5 (E8.5) days of gestation, and embryos were removed by microdissection. Real-time reverse transcriptase-mediated quantitative PCR was employed to detect all known isoforms of glucose and fatty acid transporters, using previously published (Kappen et al., 2019) primer sets. Of the glucose transporters surveyed, expression of Slc2a2/Glut2 and Slc2a7/Glut7 was undetectable by RT-PCR in any condition at either time point examined; absence of Slc2a4/Glut4 expression in embryos was also reported before (Hogan et al., 1991). As shown in Figure 2, Slc2a1/Glut1, Slc2a3/Glut3, and Slc2a12/Glut12 exhibited the highest levels of expression at E7.5 and E8.5, while Slc2a13/Glut13, Slc2a10/Glut10, and Slc2a9/Glut9 had the lowest expression. Among the genes encoding proteins involved in fatty acid transport, Slc27a4/Fatp4 displayed the highest expression level in decidua, and Slc27a5/Fatp5 the lowest level. Of the carnitine-palmitoyl transferases, isoforms 1a and 2 were most prominently expressed at all stages and conditions tested.

Notably, there were no significant differences in expression level between time points. Interestingly, there was also no evidence for differences in nutrient transporter expression between normoglycemic pregnant dams of the two different mouse strains, NOD and FVB (Figure 2, compare solid and striped color bars). Of particular relevance for nutrient transport in diabetic pregnancies is the lack of significant differences when decidua from pregnancies of normoglycemic dams (black bars) are compared to decidua samples from diabetic pregnancies (red bars); equivalence of nutrient transporter expression between the metabolic states was found in both the genetic (NOD) as well as the pharmacological (FVB) diabetes model. Taken together, our results support the conclusion that nutrient transporter expression is not altered by maternal metabolic disease. Even though we cannot exclude the possibility that protein levels and transport activities may differ, our findings reveal that the potential for nutrient transport to the embryo is not impaired at the transcriptional level, based upon assays of whole decidua (minus the embryos).

**FIGURE 3:**
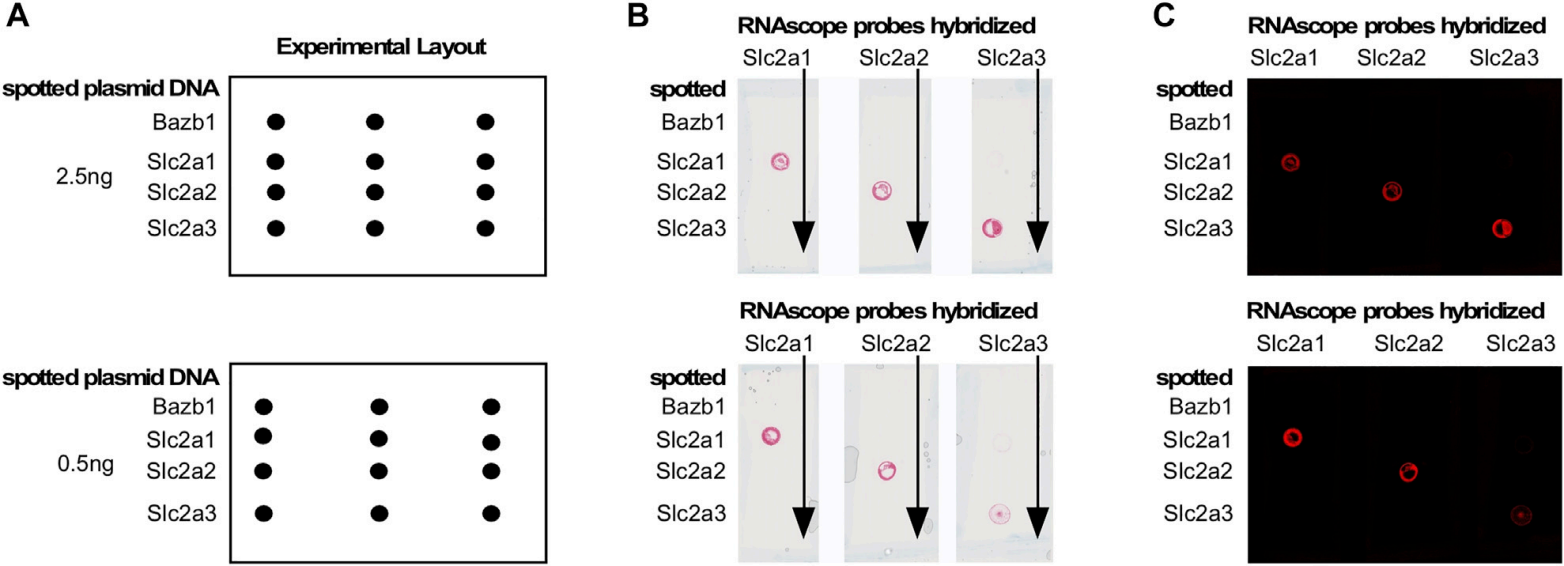
Validation of RNAscope probes for Glucose transporters. Target cDNAs in linearized form were spotted onto a glass slide as shown in the schematic in Panel **(A)**. Panel **(B)**: RNAscope probes were hybridized to all spots in a given column; probe binding (red signal) was only observed at the spot containing the cognate cDNA. Panel **(C)**: Image of the result in Panel **(B)**, which reveals the signal as red fluorescence. No cross-reactivities were observed, demonstrating exclusive specificity of each RNAscope probe for its target.

To assess whether maternal diabetes altered the cellular distribution of nutrient transporter transcripts within each deciduum, we performed RNAscope *in situ* hybridization assays on serial sections through entire implantation sites. The presence of embryos in these samples also enabled us to detect nutrient transporter expression in the early embryo prior to formation of a placenta. For these experiments, we first validated the specificity of target detection by our probes, particularly among the most highly expressed glucose transporters, Slc2a1/Glut1, and Slc2a3/Glut3, compared to Slc2a2/Glut2. Figure 3 shows hybridization of Slc2a/Glut transporter family RNAscope probes to linearized cDNAs that were spotted onto glass slides. Specific reactivity for each respective RNAscope probe was found only to its cognate cDNA, demonstrating absence of any cross-reactivity, and implying that all glucose transporter RNAscope probes used were highly specific. We then employed these probes for hybridizations to tissue sections of entire decidua, containing the conceptus as well.

**FIGURE 4:**
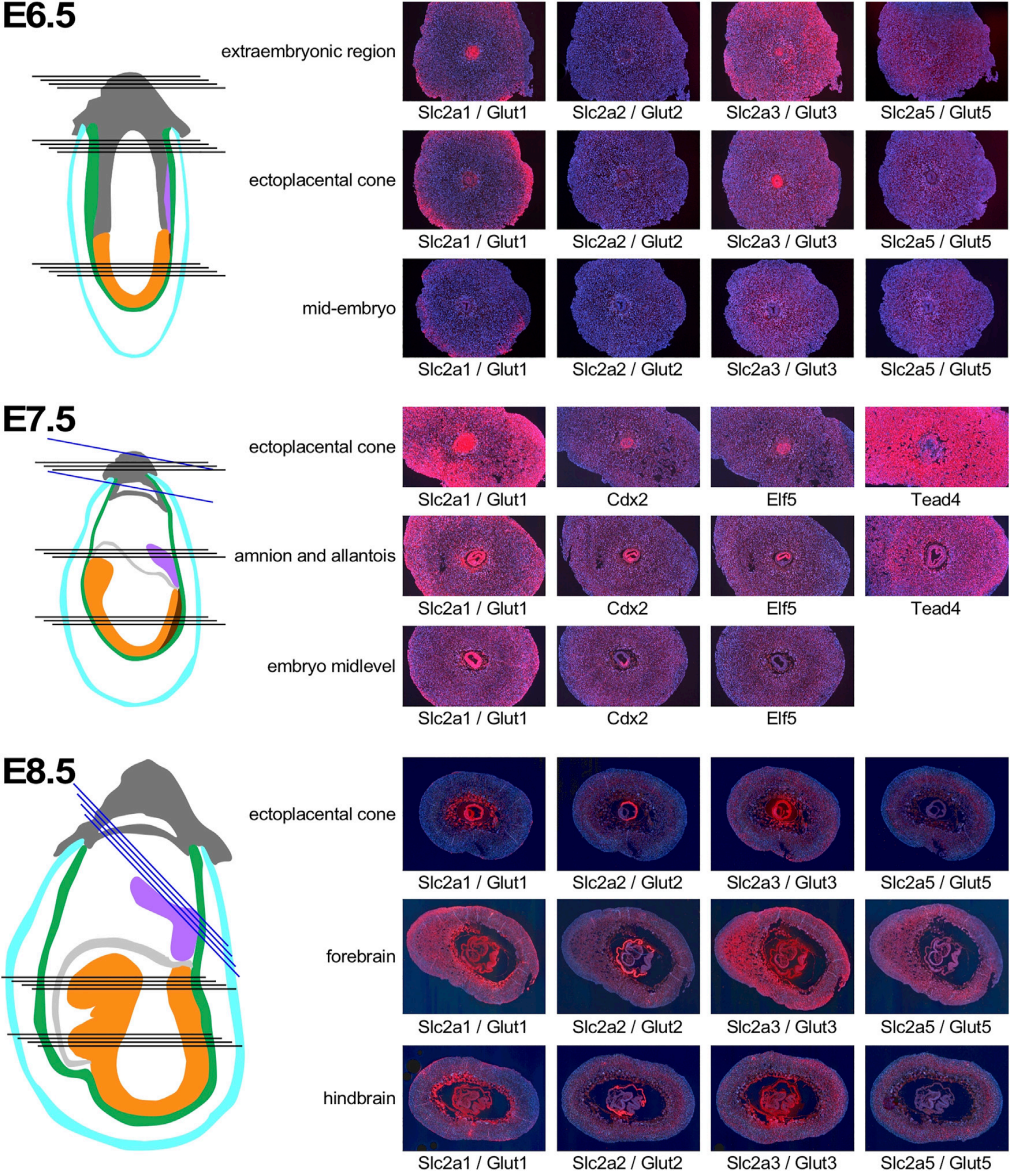
Expression of Glucose transporter genes in early pregnancy. Decidua from pregnant females of the NOD mouse strain were isolated at various time points of pregnancy, and serial sections were produced. Adjacent sections from different anatomical locations were hybridized with RNAscope probes for the respective nutrient transporters. Schematic representations indicate the orientation of sections through tissues of the implantation sites: dark grey: extraembryonic cells and ectoplacental cone; turqoise: pariental yolk sac; green: visceral yolk sac, purple: allantois; light grey: amnion; orange: embryo proper; brown: primitive streak. Black lines indicate sections produced from the same implantation site, blue lines indicate sections from a different sample for the Tead4 hybridization at E7.5, and the ectoplacental cone sections at E8.5. Where possible, embryo sections are oriented with anterior (head) to the left.

In normoglycemic females of NOD strain at 6.5 days of gestation, Slc2a1/Glut1 is expressed in the decidual tissue, particularly in outer cells, and in the extraembryonic tissue (Figure 4). Within the embryo, some signal for Slc2a1/Glut1 is detectable at this time point, mostly in neuroectodermal cells. Slc2a2/Glut2 is only weakly expressed in the deciduum, and undetectable in the embryo proper. The strongest signal is found for Slc2a3/Glut3, which is expressed throughout the deciduum, and markedly in the extraembryonic tissue, but only at moderate levels in the embryo. Slc2a5/Glut5 is also weakly expressed throughout the implantation site at this developmental stage. By E7.5, Slc2a1 expression becomes more prominent in deciduum, and extraembryonic as well as embryonic tissues display very strong expression. For comparison, the transcription factor Cdx2 exhibits moderate expression in some decidual cells, strong expression in extraembryonic tissue, and within the embryo is restricted to the posterior region and primitive streak, consistent with published data (Bialecka et al., 2012). Expression of the Ets-like transcription factor Elf5 is also readily detectable in extraembryonic tissue (Donnison et al., 2005), but absent from the embryo. Transcription factor Tead4 (Yagi et al., 2007) is very strongly expressed throughout the deciduum, with less intense signal displayed in extraembryonic cells. At E8.5, Slc2a1/Glut1 exhibits strongest expression in the ectoplacental cone and trophoblast giant cells, and throughout the embryo, where neuroepithelial cells display stronger expression than mesodermal derivatives. The only signal found for expression of Slc2a2/Glut2 is localized in the visceral yolk sac surrounding the embryo, but not in other extraembryonic cells, nor the embryo proper. At this time point, Slc2a3/Glut3 is strongly expressed in decidual cells, in cells surrounding the blood lagoons, in the parietal and visceral yolk sac, and in embryonic surface ectoderm. While neuroectoderm also exhibits signal for Slc2a3/Glut3, signals from mesoderm are rather weak. These assays show that each of the glucose transporters has a distinct pattern of expression in the embryo and surrounding tissues, with some overlap of Slc2a1/Glut1 and Slc2a3/Glut3 in neuroepithelial cells.

**FIGURE 5:**
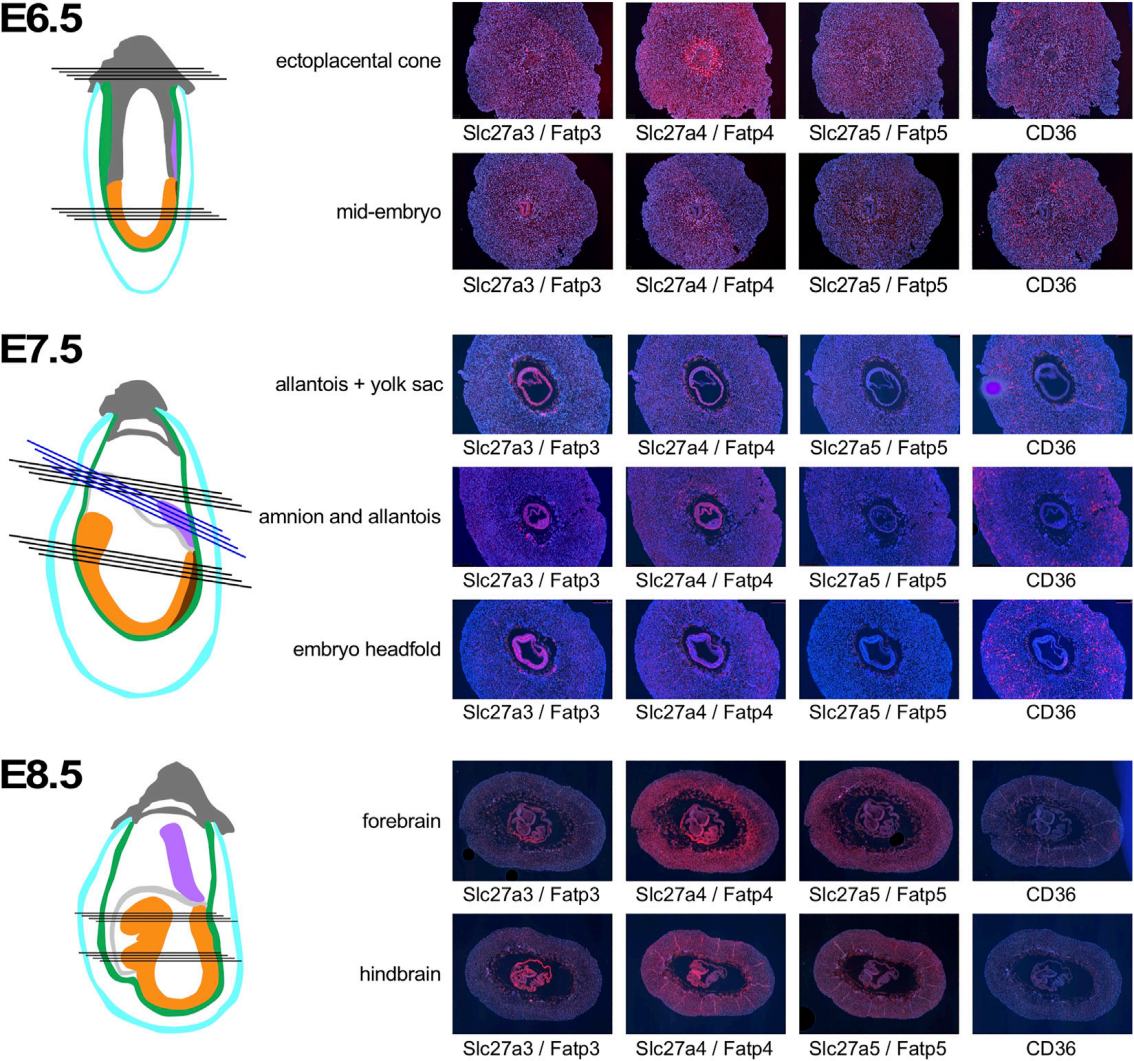
Expression of Fatty acid transporter genes in early pregnancy. Decidua from pregnant females of the NOD mouse strain were isolated at various time points of pregnancy, and serial sections were produced. Adjacent sections from different anatomical locations were hybridized with RNAscope probes for the respective nutrient transporters. Schematic representations indicate the orientation of sections through tissues of the implantation sites: dark grey: extraembryonic cells and ectoplacental cone; turqoise: pariental yolk sac; green: visceral yolk sac, purple: allantois; light grey: amnion; orange: embryo proper; brown: primitive streak. Black lines indicate sections produced from the same implantation site, blue lines indicate sections from a different sample for amnion and allantois at E7.5. Where possible, embryo sections are oriented with anterior (head) to the left.

Discrete patterns of expression were also evident for the fatty acid transporters (Figure 5). At E6.5, Slc27a3/Fatp3 appeared uniformly distributed in the decidua, with some signal from extraembryonic tissue in the ectoplacental cone. Slc27a4/Fatp4 exhibited strong expression in the deciduum around the conceptus, and also in the ectoplacental cone. Slc27a5/Fatp5 expression signals were weaker in decidual tissue. In the embryo proper, stronger signal was only evident for Slc27a3/Fatp3, while Slc27a4/Fatp4, and Slc27a5/Fatp5 displayed moderate expression. Strong signals for CD36 were absent from ectoplacental cone or embryo proper, but obvious in the deciduum, where the spatial distribution further away from the conceptus -when compared to the Slc27a family members- suggests that CD36 may be expressed by a distinct cell type. By E7.5, the Slc27a3/Fatp3- and Slc27a4/Fatp4-specific probes reveal strong expression in the embryo and yolk sac, with weaker expression of Slc27a4/Fatp4 in neuroepithelium. Appreciable signals are displayed in decidual tissue for Slc27a3/Fatp3 and Slc27a4/Fatp4, but are almost absent for Slc27a5/Fatp5. CD36 expression remains at a distance from the conceptus, and as before, the distribution appears different than for the Slc27a family transcripts. In the embryo proper, CD36 signal is appreciable in the posterior mesoderm, and undetectable in neuroepithelium. At E8.5, Slc27a3/Fatp3 expression is detectable most strongly in trophoblast giant cells, the visceral yolk sac, and embryonic mesoderm. Slc27a4/Fatp4 exhibits signals throughout the deciduum, including trophoblasts; strongest expression is found in the visceral yolk sac and, in the embryo, neuroepithelium displays markedly stronger expression than mesoderm. Slc27a5/Fatp5 signals are generally weak, with moderate but widespread expression in embryonic cells. CD36 expression appears much diminished in deciduum by E8.5, a few cells in the yolk sac display moderate signals, as do some cells in the region of the developing heart. Thus, similar to the glucose transporter gene family, genes encoding fatty acid transporters exhibit distinct levels and patterns of expression in deciduum, extraembyonic tissues and the developing embryo.

**FIGURE 6:**
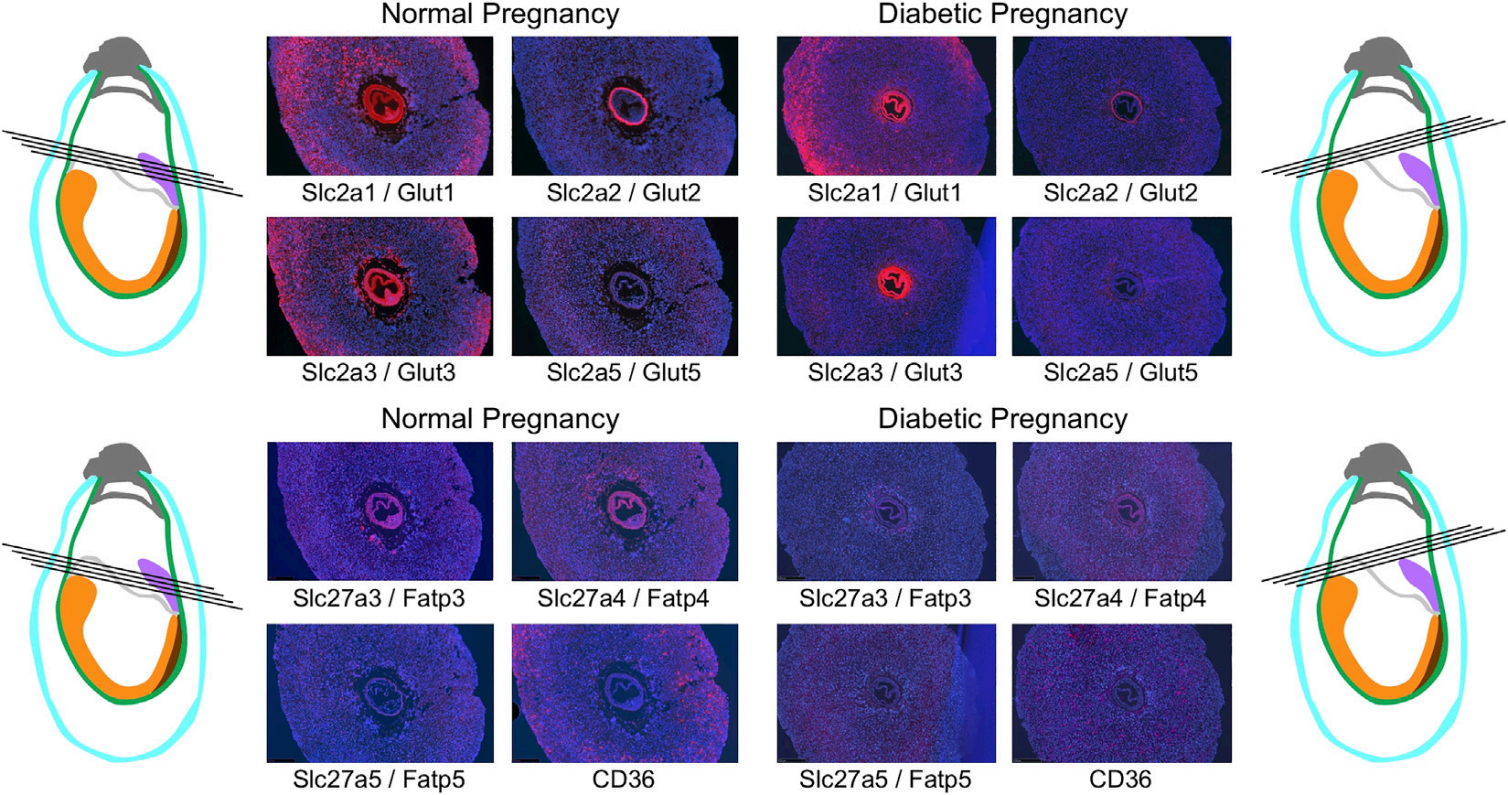
Comparison of nutrient transporter expression in decidua of normal and diabetic pregnancies at E7.5. Decidua from normal and diabetic pregnant females of the NOD mouse strain were isolated at E7.5, and serial sections were produced; their orientations are indicated by black lines. The color code for the schematic representations is the same as for Figures 4, 5.

**FIGURE 7:**
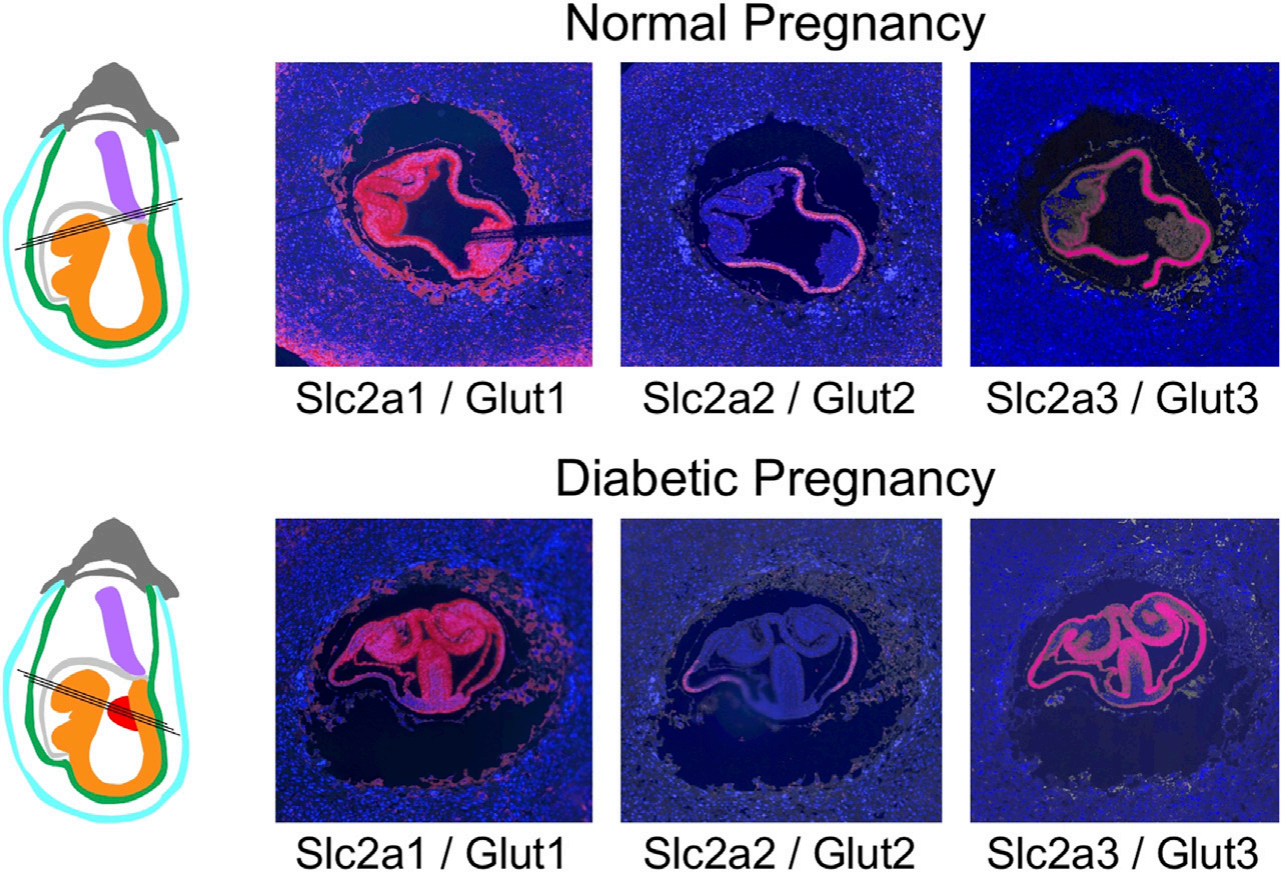
Comparison of glucose transporter expression in decidua of normal and diabetic pregnancies at E8.5. Decidua from normal and diabetic pregnant females of the NOD mouse strain were isolated at E8.5, and serial sections were produced; their orientations are indicated by black lines. The color code for the schematic representations is the same as for Figures 4–6, with the addition of ectopic tissue protrusions (red) that are specifically observed in a fraction of embryos in NOD diabetic pregnancies. These protrusions from the embryonic midline encompass embryonic mesodermcovered by neuropeithelium, and those embryo-derived cells display the same nutrient transporter expression patterns as normal embryonic mesoderm and neuroepithelium.

The general expression patterns are also evident in concepti from diabetic pregnancies (Figure 6): at E7.5, deciduum and extraembryonic tissues are positive for Slc2a1/Glut1 expression, Slc2a3/Glut3 expression is also found in deciduum and extraembryonic tissues, Slc2a5/Glut 5 expression is undetectable, and expression of Slc2a2/Glut2 is strictly limited to the visceral yolk sac, and absent from the embryo. Likewise, Slc27a3/Fatp3 and Slc27a4/Fatp4 expression is detected in extraembryonic tissues, Slc27a5/Fatp5 signals are very weak, and CD36 expression displays a unique pattern in the deciduum. No consistent differences were observed between conception sites from normal and diabetic pregnancies when additional serial sections were inspected (data not shown). At E8.5 (Figure 7), strong expression of Slc2a1/Glut1 is evident in the outer region in trophoblast cells surrounding the implant, the visceral yolk sac, and the embryo proper with stronger signals in neuroepithelium compared to mesodermal cells. Slc2a2/Glut2 is only expressed in visceral yolk sac, not in the cells of the embryo proper. Slc2a3/Glut3 exhibits highest expression in neuroepithelium and endoderm, and only low signal in mesoderm. That the particular conceptus shown in Figure 7 was indeed derived from a diabetic pregnancy is confirmed by the presence of a protrusion in the posterior region of the embryo, a feature that we only ever found in diabetic pregnancies (Salbaum et al., 2015) and that therefore is pathognomonic for embryonic exposure to maternal hyperglycemia. Taken together, these results establish that glucose transporters display distinctive expression patterns in the gastrulation stage embryo and surrounding tissues, and that these patterns are unaltered under conditions of maternal diabetes.

**FIGURE 8:**
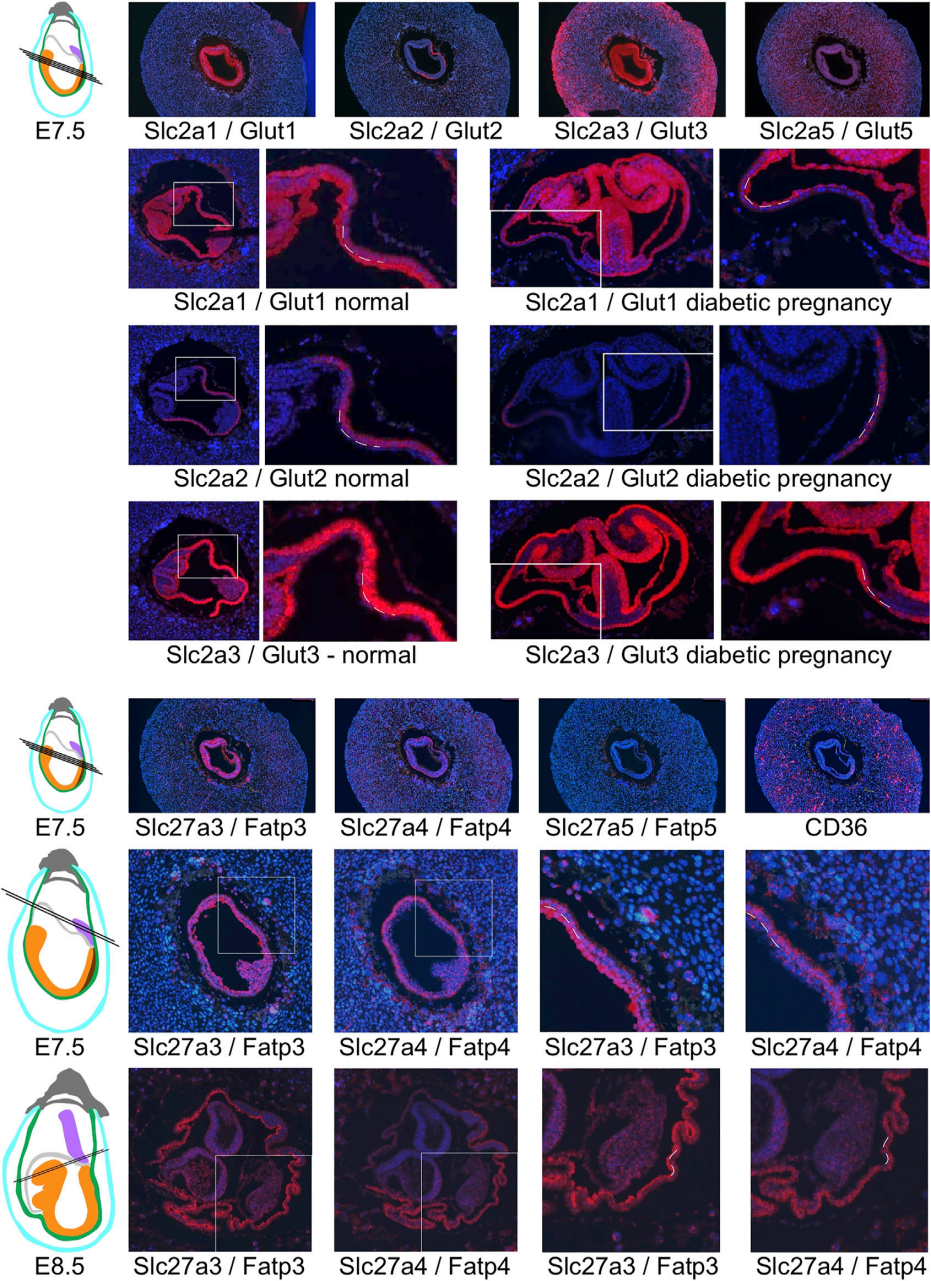
Restricted expression of nutrient transporters in visceral yolk sac cells. Glucose transporter expression is shown in decidua from normal pregnancies at E7.5, and at E8.5 from normal and diabetic pregnancies (the same sections shown in Figure 7). White frames indicate the region of magnification, and broken lines indicate the cell layers of the visceral yolk sac: endoderm is located on the outside twoards the yolk sac cavity, with the mesodermal layer of the visceral yolk sac facing the embryo. Slc2a1/Glut1 expression is prominent in the mesodermal layer, while Slc2a3/Glut3 is expressed more strongly in the endoderm layer, and Slc2a2/Glut 2 expression is restricted to visceral yolk sac endoderm. Of the fatty acid transporters, Slc27a5/Fatp5 and CD36 were not specifically detected in visceral yolk sac at E7.5 or E8.5 (not shown), while Slc27a3/Fatp3 and Slc27a4/Fatp4 display reciprocal predominance in the mesodermal and endodermal layers of the yolk sac, respectively.

Particularly intriguing was the restricted localization of Slc2a2/Glut2 in visceral yolk sac, where the other two glucose transporters are also expressed. This prompted us to investigate the cellular localization of glucose and fatty acid transporter expression in yolk sac in detail (Figure 8): Slc2a1/Glut1 exhibits stronger signal in the cell layer facing the embryo; this cell layer is known to be of mesodermal origin (Yoder et al., 1994; Zohn and Sarkar, 2010). In contrast, the cell layer facing the deciduum displays strong expression of Slc2a3/Glut3, consistent with earlier findings (Smith and Gridley, 1992). This cell layer is known to be composed of endodermally derived cells that are highly endocytic, they are involved in uptake of nutrients from the maternal circulation and yolk sac cavity (Zohn and Sarkar, 2010). Intriguingly, these endodermal cells are the only sites of signals for Slc2a2/Glut2 expression at early developmental stages. Our results thus implicate Slc2a3/Glut3, and to a lesser extent -based on weaker signal intensity- Slc2a2/Glut2, as the transporters predominant in the uptake of glucose, while Slc2a1/Glut1, by virtue of its expression in the mesodermal component of the yolk sac, would be involved in the delivery of nutrients to the embryo. Distinct localization in the mesodermal and endodermal compartments of the yolk sac was also evident for the fatty acid transporters (Figure 8), at E7.5 and E8.5. The strongest signals for Slc27a3/Fatp3 were found in mesodermal cells of the yolk sac, while the endodermal layer displayed predominantly positivity for Slc27a4/Fatp4. At E8.5, when both genes are also expressed in the embryo, the reciprocal allocation to mesodermal and endodermal cells is maintained in the visceral yolk sac.

**FIGURE 9:**
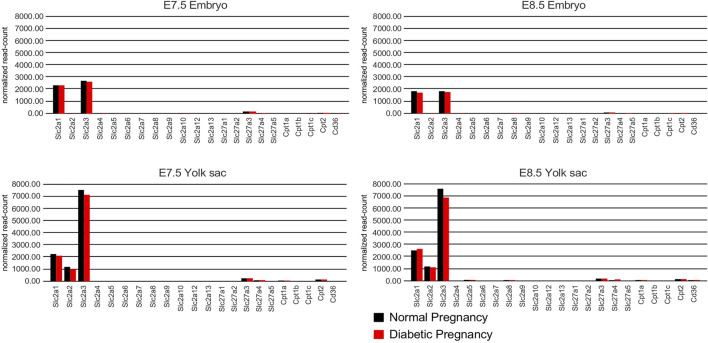
RNA sequencing results from embryos and corresponding visceral yolk sacs isolated from normal and diabetic pregnancies of the NOD strain at E8.5. The *Y*-axis depicts the number of unique molecular identifier (UMI) sequence reads for each gene. Bars represent the average of *n* = 17 pairs of embryo and corresponding yolk sac for each group at the 5 and 6 somite stage, and *n* = 16 sample pairs for each group at the 7–9 somite stage. Bars are absent for genes that were undetectable or yielded a read number smaller than 10. No statistically significant differences were found between the averages for samples from normal and diabetic pregnancies, except for Slc2a4 detection in 5/6 somite stage embryos (see Supplementary Table S1 for numerical data).

Overall, the results of our histological assays are consistent with the RT-PCR measurements of nutrient transporter expression in decidua, insofar as we did not observe significant expression differences between samples derived normal and diabetic pregnancies. However, the RNAscope assays of expression in the visceral yolk sac may not adequately reflect quantitative differences of transporter expression levels. Therefore, to assess potential effects of maternal diabetes on transporter expression levels in the visceral yolk sac specifically, we interrogated a next-generation RNA sequence dataset (J.M. Salbaum et al., manuscript in preparation) derived from embryos and corresponding yolk sacs that were isolated at E8.5 from normal and hyperglycemic pregnancies in the NOD strain (Figure 9; Supplementary Table S1). In embryos with 5 or 6 somite pairs, the highest levels of expression were observed for Slc2a3/Glut3, followed by Slc2a1/Glut1, and the fatty acid transporters Slc27a3/Fatp3 and Slc27a4/Fatp4. The same genes were prominently expressed in embryos at the 7–9 somite stages. In the yolk sac, Slc2a3/Glut3 was the highest expressed glucose transporter, followed by Slc2a1/Glut1 and Slc2a2/Glut2, at both developmental stages. Expression of Slc27a3/Fatp3 and Slc27a4/Fatp4, as well as Cpt2, was also consistently detected. Notably, exposure to maternal diabetes did not significantly alter expression levels of the nutrient transporters tested here, although the already low-level expression of Slc2a4/Glut4 observable in embryos at the 5–6 somite stage was further reduced in diabetic conditions (see Supplementary Table S1 for numerical data). However, no expression was detectable in NOD embryos at the 7–9 somite stages regardless of metabolic condition (Supplementary Table S1), nor in diabetes-exposed FVB embryos at later stages (Pavlinkova et al., 2009), leaving the biological relevance of the finding to be investigated. Also notable are the very low counts for Slc2a5 in early stage embryo and yolk sac samples, consistent with the absence of signals in the RNAscope assays. The detection of Slc2a2/Glut2 in embryos by RNA sequencing is subject to the caveat that it is very difficult to remove the visceral endoderm completely, and thus, low counts for Slc2a2/Glut2 could derive from a few contaminating yolk sac cells. Indeed, Slc2a2/Glut2 expression is highly prominent in yolk sac samples, irrespective of metabolic condition, at both developmental stages.

Furthermore, 231 and 232 known nutrient transporter-encoding genes were measurable by next-generation sequence assays –with the majority of genes displaying very low count number– in visceral yolk sac associated with embryos with 5/6 and 7–9 somite pairs, respectively. Expression of 228 transporters was detected at both developmental stages, but there were no significant differences between normal or diabetic conditions (data not shown). Taken together, our quantitative and qualitative results do not provide evidence of major changes in the amounts or patterns of expression of nutrient transporters in early embryos, associated yolk sacs, or their decidual environment, that could be explained by exposure to maternal diabetes. Thus, –at the transcriptional level– nutrient transporter expression is unaltered by maternal metabolic disease and therefore unlikely to account for the increased susceptibility for birth defects and adverse outcomes in diabetic pregnancies.

## Discussion

In this study, we provide evidence on the expression of nutrient transporters in early post-implantation mouse development. We show that different nutrient transporters have distinct patterns of cell-and tissue-specificity in decidua as well as the conceptus. Thus, our results confirm earlier observations made with radioactive *in situ* hybridization studies (Smith and Gridley, 1992). Our results further highlight which nutrient transporters are undetectable or expressed at very low levels, making it unlikely that they play a functional role in early development in the mouse, or in developmental abnormalities associated with diabetic pregnancies.

A particular strength of our approach is the inclusion of two inbred mouse strains, FVB and NOD, in which maternal diabetes arises from pharmacological manipulation, or genetic predisposition, respectively. While the FVB-STZ model produces profound hyperglycemia, the NOD model additionally exhibits auto-immune and inflammatory processes that are also characteristic of human type I diabetes (Atkinson and Leiter, 1999). The results for normoglycemic pregnancies in both mouse strains are consistent, in terms of patterns as well as levels of expression of the nutrient transporters. We also investigated the effects of maternal diabetes/hyperglycemia on nutrient transporter expression in both strains. Although the metabolic derangements in the pregnant diabetic dams involve elevated levels of glucose and lipids in the maternal circulation, nutrient transporter expression under these conditions was similar to normoglycemic pregnancies. There were no statistically significant differences in the profiles or levels of transporter expression by metabolic condition. Thus, the availability of glucose and lipids does not affect levels of expression of the nutrient transporters. Yet, because our RT-PCR and RNA sequencing assays only detected steady-state mRNA levels, this conclusion does not exclude the possibility that protein levels might differ, or that the rates of activity and overall extent of nutrient transport –and thus nutrient availability to the embryo– could be altered in pregnancies affected by maternal diabetes and hyperglycemia. Earlier reports on higher glucose content in mouse embryos exposed to maternal hyperglycemia (Neubert et al., 1971; Cockroft and Coppola, 1977; Rashbass and Ellington, 1988; Ellington, 1997) and of elevated fatty acid uptake in fetal rat liver in diabetic rats (Goldstein et al., 1985) provide some support for this notion, although measurements in these reports were performed at later stages of development than reported here. Our assays at E7.5 and E8.5 do not provide evidence that nutrient excess alters transcription or mRNA stability of nutrient transporters.

Intriguingly, our *in situ* hybridization results demonstrate that nutrient transporters are expressed with distinct tissue- and cell-type specific distribution in post-implantation embryos and extra-embryonic tissue, particularly the visceral yolk sac. Strong expression in the endodermal cell layer of the yolk sac was evident for Slc2a3/Glut3 and Slc27a4/Fatp4, and these cells are the sole sites of Slc2a2/Glut2 detection at all developmental stages examined. In contrast, Slc2a1/Glut1 and Slc27a3/Fatp3 were prominently expressed in the mesodermal cell layer. In addition, the embryonic sites of expression, although overlapping between some transporters, were specific to each gene. The distinct cell– and tissue–specific patterns indicate that transcriptional regulatory mechanisms, and specific transcription factors involved, are likely distinct for each nutrient transporter gene, and in the particular cell types where they are expressed. For example, the transcription factor HNF4α has been shown to be required in the visceral endoderm for uptake of nutrients (Chen et al., 1994), and for transcription of Apo-B, which was subsequently demonstrated to mediate lipid transport in the visceral endoderm (Farese et al., 1995). HNF4α also regulates expression of Folate Receptor 1 in these cells; endodermal cells in the visceral yolk sac are the only site of expression of this folate transporter at E7.5 (Salbaum et al., 2009).

These findings prompt the conclusion that the beneficial effect of folic acid supplementation for prevention of neural tube defects in diabetic pregnancies is mediated by uptake of this nutrient in the visceral endoderm. It has been shown that supplementation of folic acid to the pregnant dam is able to overcome Folate Receptor 1–mediated transport defects even in pregnancies where the yolk sac is deficient for Folate Receptor 1 (Piedrahita et al., 1999; Zhu et al., 2007), thus preventing neural tube and heart defects in supplemented FolR1-deleted mouse embryos. This observation suggests that supplementation of appropriate metabolites for transport by Slc2a3/Glut3 and Slc27a4/Fatp4 in visceral endoderm might also have efficacy in preventing the embryonic lethality in mouse mutants with genetic deficiency of the Slc2a3/Glut3 (Schmidt et al., 2009) and Slc27a4/Fatp4 (Gimeno et al., 2003) transporters, respectively. An alternative, and also testable, hypothesis would be that micronutrients can affect the nutrient transport function in general in endodermal cells of the visceral yolk sac.

Less is known about the functional role of nutrient transporters expressed in the mesodermal layer of the visceral yolk sac. Mice with targeted deletion of Slc2a1/Glut1 exhibit embryonic lethality between E10 and E14 (Wang et al., 2006), while Slc27a3/Fatp3 knockout mice have not been generated to date. It has been argued, based upon widespread expression of genes in the conceptus that are also expressed in yolk sac mesoderm (Palis and Kingsley, 1995), that yolk sac mesodermal cells at E7.5 are “a largely undifferentiated tissue”, prior to vascular development and hematopoietic cell generation (Ogawa et al., 1999; Ogawa et al., 2001; Golub and Cumano, 2013). However targeted deletion of VEGFR/flt-1, a marker detected as restricted to visceral yolk sac mesoderm as early as E6.5 (Fong et al., 1996), demonstrates the presence of cells committed to the vascular endothelial lineage at this early stage (Fong et al., 1999). Intriguingly, the development of yolk sac mesenchyme itself is dependent on sufficient levels of VEGF (Duan et al., 2003). Reciprocal signaling between mesoderm and endodermal compartments overall has been shown to be required for proper development of the mouse yolk sac (Rhee et al., 2013). How these developmental pathways are influenced by nutrient transport functions in the visceral yolk sac cell layers remains to be investigated.

The original motivation for this study was to test whether the altered nutrient availability in pregnancies affected by maternal diabetes is associated with altered expression of nutrient transporters. Neither the quantitative RT-PCR data nor the *in situ* hybridization results provide evidence for this scenario, and additional data from RNA sequencing experiments are consistent with these results. Nevertheless, our findings have relevance to understanding the pathogenesis of structural birth defects and the elevated risk for future metabolic disease in the offspring of diabetic pregnancies from five perspectives:

1) It was proposed that embryos suffer the effects of hyperglycemia due to their expression of the high capacity glucose transporter Slc2a2/Glut2, because when embryos are homozygous for the deletion allele of Slc2a2/Glut2 (Guillam et al., 1997), the rate of neural tube defects is reduced compared to wildtype (Li et al., 2007). Our results, however, show that Slc2a2/Glut2 is not expressed in the embryo proper, but in the visceral yolk sac endoderm, which is embryo-derived, but considered an extraembryonic tissue at the time when Slc2a2/Glut2 is expressed there (Bielinska et al., 1999; Kinder et al., 1999; Zohn and Sarkar, 2010). Given that this is the only site of Slc2a2/Glut2 expression at post-implantation stages just prior to neural tube closure, the protective effect of Slc2a2/Glut2 deletion then can only be mediated by cells of the visceral endoderm, rather than within those cells of the embryo itself that are involved in neural tube closure. Absence of Slc2a2/Glut2 expression in visceral yolk sac of knockout mutant embryos may reduce glucose transport to the mutant embryo and thus “normalize” the nutritional environment for mutants, but this would not apply for the yolk sacs associated with heterozygous or wildtype embryos, which would continue to be susceptible to the detrimental effects of maternal hyperglycemia.
2) By far the highest expression of any glucose transporter in normal and diabetic implantation sites, according to signal intensity in the *in situ* hybridizations and supporting RNA sequencing results, is that of Slc2a3/Glut3 in the visceral endoderm. It is therefore a plausible possibility that Slc2a3/Glut3–mediated glucose uptake is increased under conditions of hyperglycemia and that excess glucose uptake leads to changes in the visceral endoderm cells themselves. This tenet is implicit to the “yolk sac hypothesis” for pathogenesis of pregnancy hyperglycemia–related complications (Reece et al., 1994b). Reports of visceral yolk sac abnormalities at the structural (Pinter et al., 1986b) and biochemical (Pinter et al., 1986a; Reece et al., 1989) levels in experimental animal diabetic pregnancies provide support for this model. Our results now provide evidence that such abnormalities are not accompanied by transcriptional dysregulation, but suggest functional differences in nutrient transport and metabolism in visceral yolk sac cells. An important practical corollary is that investigation of causal mechanisms for hyperglycemia-induced complications then should be less focused on the embryo itself but on the role of the visceral endoderm as primary target of the exposure. Analogously, the specific and high-level expression of Slc27a4/Fatp4 in visceral endoderm prompts the hypothesis that hyperlipidemia may affect cells in the visceral yolk sac directly.
3) Yet, the foregoing considerations cannot satisfactorily explain a very consistent observation in animal models of diabetic pregnancy, namely that specific tissues and structures in the exposed embryos and fetuses are preferentially affected: neural tube closure, caudal structures, heart, and skeletal development. Our *in situ* hybridizations reveal that embryonic mesoderm, from which the heart precursors derive (Rosenquist, 1970; Lawson and Pedersen, 1992; Kappen and Salbaum, 2009), exhibits lower glucose and fatty acid transporter expression levels than, for example, neuroepithelium. Also, the broad expression of the glucose and fatty acid transporters within the neuroepithelium cannot explain the appearance of neural tube closure defects at distinct sites in the CNS and spinal cord. Thus, the factors making particular cell types, tissues, and locations especially vulnerable to nutrient excess -and subsequent defective morphogenesis- remain to be identified. Likewise, if altered visceral endoderm transport under diabetic conditions reduces the supply of particular nutrients and metabolites to the embryo, the causes for selective effects on particular tissues and locations are not easily attributable based on distribution and levels of nutrient transporter expression in the embryo proper.
4) The visceral endoderm localization of Folate Receptor 1 expression (Salbaum et al., 2009) implies the prospect that supplementation of folic acid might act directly on the visceral endoderm. We showed previously that folic acid supplementation reduces the incidence of neural tube defects in diabetic pregnancies of the NOD mouse strain (Salbaum et al., 2015). For the Streptozotocin-induced diabetes model, folic acid was also shown to reduce defects, alone (Wentzel et al., 2005; Oyama et al., 2009) and in combination with Vitamin E (Gareskog et al., 2006). Deletion of the transporter for Vitamin E, Scavenger receptor B1, is associated with neural tube defects (Santander et al., 2013), which can be rescued by Vitamin E supplementation (Santander et al., 2018). SR-B1 is expressed in visceral endoderm (Hatzopoulos et al., 1998; Santander et al., 2013) (and our own unpublished data), highlighting a possible role of visceral endoderm in the beneficial effects of vitamin E supplementation in diabetic pregnancies. Further support for this notion comes from the finding that Glucose Transporters Glut1 and Glut3, the genes encoding for which we show to be expressed in the visceral yolk sac mesoderm and endoderm, respectively, are active transporters for dehydroascorbic acid (Rumsey et al., 1997), the oxidized form of Vitamin C. It is tempting to speculate that the high glucose supply in diabetic pregnancies might outcompete Vitamin C transport to the embryo by Glut3 and Glut1, a scenario that is amenable to experimental testing. Whether alternative transport mechanisms may be engaged when deficient nutrients are supplemented in excess also remains to be determined. Even though our results do not provide evidence that expression levels of nutrient transporters are altered by nutrient excess, it is conceivable that transporter activities and the extent of nutrient transport by the visceral yolk sac could be affected. It will also be interesting to investigate whether these nutrients play roles in the visceral endoderm and mesodermal cells themselves, possibly by regulating their metabolism and energy available for nutrient transport more generally.
5) Finally, the cell-type-specific expression of nutrient transporters Slc2a1/Glut1 and Slc27a3/Fatp3 in mesoderm of the visceral yolk sac also raises implications for long-term sequelae of *in utero* exposures, often termed “developmental programming”, in line with the concept of “developmental origins of health and disease” (DOHaD) (Mass and Gentek, 2021). Mesodermal cells of the visceral yolk sac give rise to precursors that colonize first -as microglia- the developing brain, and later -as tissue-resident macrophages- other organs, where they persist into adulthood (Wu and Hirschi, 2020). Because tissue surveillance by the innate immune system is critical to the health of many organs (Elsaid et al., 2020), any exposure that affects these yolk sac derived macrophages, pre- or postnatally, can potentially alter their function in the health of one or more organs. Given that macrophages are powerful elicitors of inflammation when they encounter infection or injury (Cox et al., 2021), they control the immune status of adult tissues, and may thus contribute to long-term susceptibility for chronic diseases that are characterized by increased inflammation, such as insulin resistance, diabetes, obesity, and cardiometabolic diseases. The potential pathophysiological roles of nutrient transporter expression in mesodermal cells of the yolk sac for the generation and function of long-lived tissue-resident macrophages remain to be investigated.

## Data Availability

The original contributions presented in the study are included in the article/Supplementary Material, further inquiries can be directed to the corresponding author. The next generation sequencing data that were analyzed for this study are deposited in the Gene Expression Omnibus (GEO) repository, accession number GSE197396.

## Supplementary Material

Supplementary Table 1 Expression of nutrient transporters as measured by RNA next-generation sequencing. RNA was extracted from corresponding samples of embryo and yolk sac, from normal and diabetic pregnancies in the NOD strain. All samples were processed individually. Embryos had been staged according to the number of somite pairs present, and sequencing results were grouped by developmental stage of 5–6 somite pairs or 7–9 somite pairs, respectively. This table lists the numerical data depicted in graphical form in Figure 9.
