# Supplementary material for: Nutrient Transporter Gene Expression in the Early Conceptus—Implications From Two Mouse Models of Diabetic Pregnancy
Source: Front Cell Dev Biol. 2022 Apr 11;10:777844. doi: 10.3389/fcell.2022.777844 (PMC9035823; doi:10.3389/fcell.2022.777844)
Supplement: Supplementary file 1 [file DataSheet1.PDF]

Supplemental Table 1

Embryo	Pregnancy Control	Diabetic	
5,6 somite pairs	n=17	n=17	adj. P-value
Slc2a1	2329.33	2319.58	0.9823
Slc2a2	21.23	19.88	0.9311
Slc2a3	2687.71	2626.09	0.8672
Slc2a4	22.14	13.25	0.0054
Slc2a5	7.48	7.74	0.9649
Slc2a6	nd	nd	
Slc2a7	nd	nd	
Slc2a8	19.89	19.84	0.9797
Slc2a9	nd	nd	
Slc2a10	14.01	9.81	0.2124
Slc2a12	nd	nd	
Slc2a13	nd	nd	
Slc27a1	nd	nd	
Slc27a2	nd	nd	
Slc27a3	125.19	118.62	0.7808
Slc27a4	48.81	43.82	0.3737
Slc27a5	nd	nd	
Cpt1a	nd	nd	
Cpt1b	nd	nd	
Cpt1c	27.37	25.46	0.7749
Cpt2	57.35	56.00	0.8487
Cd36	17.92	20.56	0.5933

Embryo	Pregnancy Control	Diabetic	
7-9 somite pairs	n=16	n=16	adj. P-value
Slc2a1	1787.19	1669.36	0.4623
Slc2a2	11.74	23.35	0.2702
Slc2a3	1782.35	1710.57	0.6415
Slc2a4	16.19	12.39	0.1734
Slc2a5	7.03	8.82	0.3709
Slc2a6	nd	nd	
Slc2a7	nd	nd	
Slc2a8	17.19	21.53	0.2385
Slc2a9	nd	nd	
Slc2a10	14.76	17.35	0.5728
Slc2a12	nd	nd	
Slc2a13	nd	nd	
Slc27a1	nd	nd	
Slc27a2	nd	nd	
Slc27a3	87.31	88.21	0.9666
Slc27a4	32.14	33.55	0.9044
Slc27a5	nd	nd	
Cpt1a	nd	nd	
Cpt1b	nd	nd	
Cpt1c	23.10	23.84	0.9487
Cpt2	47.43	56.68	0.0637
Cd36	15.98	17.36	0.6125

Yolk sac	Pregnancy Control	Diabetic	
5,6 somite pairs	n=17	n=17	adj. P-value
Slc2a1	2252.47	2101.26	0.7700
Slc2a2	1181.53	991.33	0.3858
Slc2a3	7543.41	7087.84	0.7700
Slc2a4	nd	nd	
Slc2a5	7.08	5.26	0.7204
Slc2a6	nd	nd	
Slc2a7	nd	nd	
Slc2a8	6.90	6.71	0.9876
Slc2a9	nd	nd	
Slc2a10	2.92	3.29	0.9674
Slc2a12	nd	nd	
Slc2a13	nd	nd	
Slc27a1	nd	nd	
Slc27a2	nd	nd	
Slc27a3	214.79	218.80	0.9756
Slc27a4	65.45	73.65	0.8072
Slc27a5	nd	nd	
Cpt1a	6.76	11.72	0.1126
Cpt1b	nd	nd	
Cpt1c	5.17	6.77	0.6263
Cpt2	106.40	91.22	0.5278
Cd36	2.87	4.16	0.7375

Yolk sac	Pregnancy Control	Diabetic	
7-9 somite pairs	n=16	n=16	adj. P-value
Slc2a1	2511.38	2601.08	0.8869
Slc2a2	1183.43	1089.98	0.6621
Slc2a3	7597.48	6860.21	0.5272
Slc2a4	nd	nd	
Slc2a5	9.32	7.36	0.7395
Slc2a6	nd	nd	
Slc2a7	nd	nd	
Slc2a8	9.37	8.00	0.6933
Slc2a9	nd	nd	
Slc2a10	6.36	6.46	0.9798
Slc2a12	nd	nd	
Slc2a13	nd	nd	
Slc27a1	nd	nd	
Slc27a2	3.91	7.19	0.2809
Slc27a3	208.99	184.69	0.6809
Slc27a4	63.68	77.60	0.5320
Slc27a5	2.91	3.15	0.9453
Cpt1a	6.81	8.98	0.6026
Cpt1b	nd	nd	
Cpt1c	5.90	5.62	0.9096
Cpt2	130.90	121.30	0.8159
Cd36	7.67	10.23	0.6883
